# Approach to Sample Collection, Transport and Processing of Suspected or Confirmed COVID-19 Patients for Biochemistry Investigations

**DOI:** 10.31729/jnma.5705

**Published:** 2020-12-31

**Authors:** Apeksha Niraula, Basanta Gelal, Madhab Lamsal

**Affiliations:** 1 Department of Biochemistry, B.P. Koirala Institute of Health Sciences, Dharan, Nepal

**Keywords:** *biosafety measures*, *laboratory*, *specimen handling*

## Abstract

During this global pandemic period of COVID-19, the health care system is the most affected area. Health care workers including the clinicians, laboratory professional and other allied health workers pose a high threat for acquiring and transmission of the disease. Apart from the diagnosis of disease by the RT-PCR, other laboratory investigations are equally essential in disease prognosis and monitoring. This biosafety guidance is intended to provide and insight to the clinicians, nurses and laboratory professionals in handling the blood and other body fluid samples for biochemical investigations concerning the proper methods of sample collection, transport, processing and disposal. Every day, the laboratory receives samples of the patient with confirmed and suspected cases of COVID-19 patients. This pose a high threat of contamination to the health professionals and thus, a proper risk assessment and standard precautions protocol must be followed to ensure safety, minimize the risk of contamination and disease transmission.

## INTRODUCTION

The outbreak of novel corona virus COVID-19 took place in December, 2019 in Wuhan, China with further spread to other parts of the world.^1^ COVID-19 is believed to be transmitted through the respiratory tract and can induce pneumonia.^1–2^ The challenge is posed over the public health laboratories due to the ongoing outbreak as the infection is widespread and its international spread through travellers is now evident, as is spread from affected individuals. All laboratories should strictly follow the standard guidelines for sample collection, transport, processing and waste disposal. In context to COVID-19, due to the novel characteristics of the virus and its global threat, extra vigilant measures must be followed.^3^ Standard guidelines set by organizations such as CDC, WHO etc may not be strictly adapted to all the laboratories especially in the developing countries due to resource constraint. However, minimum set operational guidelines should be followed taking into considerations the optimum use of available resources with highest level of professionalism.^4^ All specimens collected for laboratory investigations must be treated as potentially infectious. With reference to COVID-19, virus yield is highest in respiratory specimens.^5–6^ However, virus may be shed in blood, other body fluids and body secretions such as saliva, broncho-alveolar lavage, blood, urine, feces, sputum, tears and semen.^5–6^ Extra safety precautions need to be taken while handling the specimens during aerosol generating procedures such as centrifugation, during analysis in autoanalyzer.^4–5,7^ Appropriate personal protective equipment PPE as determined by a detailed risk assessment should be worn by all laboratory personnel handling these specimens.^7–10^

## STEP-BY-STEP APPROACH FOR SAMPLE COLLECTION, TRANSPORT, PROCESSING OF SUSPECTED OR CONFIRMED COVID-19 PATIENTS

### Hand Hygiene

Laboratory personnel must perform proper hand hygiene after contact with potentially infected material, and before and after using PPE. Health care professionals ( HCP) should wash their hands or at least 20 seconds using soap and water or should disinfect their hands with 60-95% alcohol-based hand disinfectant. If the hands get visibly contaminated, it should be washed with soap and water.^10–12,14,13^

### Personal Protective Equipment (PPE)

COVID-19 is caused and transmitted primarily by contact or via droplet transmission due to large respiratory particles which are subjected to gravitational forces and travel only approximately one meter from the patient.^13^ Droplet or contact transmission occurs while in close contact with the patient or during the aerosol generating procedures^13^ Personal protective equipment is an important component, but only one part, of a system protecting staff and other patients from COVID-19 cross-infection.^13–14^ PPE ineptitude may contribute to nosocomial transmission of COVID-19.^15^ The usage of personal protective equipment (PPE) reduces the risk of transmission but does not fully eliminate it.^14^

Each health institutions should have their respective procedures and policies for the correct order of donning and doffing these PPE in a safe manner adhering with the standard protocols.^14^ As per the standard guidelines provided by World Health Organization (WHO),^15^ the standard order practice for donning the PPE after performing hand hygiene is gown, mask, goggles, face shield, and gloves; the order for doffing the PPE is gloves, face shield, goggles, gown and mask. The mask should be worn until the HCP leaves the contaminated area.^13,15–16^

Based on the risk of exposure dependent upon the type of activity and the transmission subtleties of the pathogen such as contact, droplet or aerosol, the use of PPE will be variable. Use of PPE should be rational and scientific.^16–17^ The HCP should minimise the PPE which should be used based on the risk of exposure (e.g. type of activity) and the transmission dynamics of the pathogen (e.g. contact, droplet or aerosol). The overuse of PPE can have a further impact on supply shortages. As per the guidelines issued by WHO, the PPE used for Laboratory Professional (LP) should be done accordingly.^16–17^ Observing the following recommendations will ensure rational use of PPE.

The recommended PPE for the LP working with the respiratory samples or aerosol generating procedures includes: FFP2 mask, Double nonsterile gloves, Long sleeved water-resistant gown, Goggles or face shield.^14^

As per our institutional guidelines, during the aerosol generating procedures like centrifugation of the blood samples and handling of the suspected and confirmed COVID-19 patient samples, we use Level II PPE which includes: Disposable Surgical Cap, N95 mask, Surgical Gown / Medical Protective Gown Goggles, Face Shield and Disposable gloves.

## SAMPLE COLLECTION

Samples sent for biochemical investigations includes blood, urine and other body fluids (Pleural, peritoneal, CSF) etc. Sample Collection can be done at clinical wards or the sample collection unit of laboratory. Sample collection of the inpatients should be done by treating doctor or nurses following the standard precautions and measures.^20^ Sample collection of the close contacts of the suspected or confirmed patients of COVID-19 should be done by technical staff or certified phlebotomist following all the standard precautions. The person withdrawing the sample of suspected or confirmed COVID-19 cases must wear the personal protective equipment as recommended by the respective institute following the national/ international guidelines. Anatomical site for arterial and venous sample collection should be same as done in other patients. However, care must be taken not to mix the blood samples with the intravenous fluids, especially in patients under iv fluids in wards. Sample collection must be done preferably by the vacuum blood collection tubes ensuring the air tight capping to prevent spillage. Person collecting the blood sample must ensure: Appropriate blood volume, Appropriate vials E.g. all samples for serum should preferably be collected in tubes containing SST clot activator, EDTA vials should be used for CBC analysis. Tube cover must be intact without any spillage or breakage of the tube. In case of spillage of the samples, the person collecting the sample should immediately remove the PPE and clean the area and the tube with 1% Sodium Hypochlorite.^17,21^ The spillage should be cleaned as soon as possible by the health attendant (helper) nearby to the laboratory area. The tube cover must be intact with the vial while sending to the laboratory.

## SAMPLE TRANSPORT

The vial containing the blood sample must be packed in a zip lock and place it in a plastic/ice box carefully. The sample to be sent to the laboratory must be collected in a batch or if feasible at a single time, and sealed in a zip lock and should be transported in the plastic or ice box securely.^18–21^ Prior information to the laboratory personnel before sample transportation from ward or collection unit is essential for the safe and timely processing of the samples and timely reporting. Specimens should be correctly labelled and accompanied by a diagnostic request form.^20–21^ Sample transport must be done following the standard precautions by the health aid of the respective ward. Sample transport by the patient attendant is not advisable as the safe transport of the samples will not be ensured. It is advisable to document the sample details after the sample is transported to the respective laboratory with the maintenance of a record. The sample must be assembled according to the registration number and details as per the investigation form and must be safely placed in the sample transport box to prevent sample spillage and distortion of the vials. The person (i.e., the health attendant) responsible for the sample transport must provide the details of the number of samples brought with their signature in the maintained register.

## SAMPLE RECEIVING

Sample should be received by the on-duty technical staff following the standard precautions.^21–22^ It is recommended that the technical staff who receives the sample should wear PPE and complete all the further steps of processing of the sample, analysis and reporting. This will ensure minimum resource utilization with maximum task. The sample box should be properly disinfected with 1% hypochlorite. If hypochlorite is not available, 62-71% ethanol or , 0.5% hydrogen peroxide could be used.^14,21–22^

The zip lock containing the sample must be opened inside a certified Class II Biological Safety Cabinet (BSC). Precautions must be taken while centrifugation of the samples by proper use of personal protective equipment (PPE), use of centrifuge safety cups; and sealed centrifuge rotors to reduce the risk of exposure to laboratory personnel. The outer surface of the sample vials must be disinfected with 70% alcohol or 0.5% sodium hypochlorite. The technical staff must wait for 30 minutes thereafter' ^14,21–22^

## SAMPLE PROCESSING

The sample containing vial must not be decapped to prevent aerosol contamination.^4–5^ Capped vial containing sample should be centrifuged with biosafety cap. Centrifugation is a crucial step which has high risk of aerosol generation. The technical staff must wear disposable Surgical Cap, N95 mask, surgical Gown / medical protective gown Goggles, face shield and disposable gloves.

While centrifuging the samples to prevent direct contamination careful measures must be followed during operation and maintenance of the centrifuge. Centrifuge should be regularly cleaned by neutral cleaning solutions (70% alcohol or alcohol-based disinfectant) applied with a soft cloth to rotors and accessories.^23^ Daily cleaning should include the interior portion of the centrifuge, the rotor chamber, and surfaces with electronic components, such as touchscreens and keypads. It is important to be aware of the different types of samples used with the centrifuge and any specific products or protocols necessary for cleaning spills. ^17,21–22,24–25^

## SAMPLE ANALYSIS

The analysis of the samples processed in the dedicated auto analyzer/ semi auto analyzer/ELISA/ CLIA/ISE must be done with PPE (disposable Surgical Cap, surgical mask, goggles, face shield and disposable gloves) for safety of the laboratory personnel. Ensure the working area to be clean and properly disinfected. Disinfection should be done with 1% Hypochlorite. Keep all the dedicated equipment (including pipettes) for sample analysis in ready to work condition. Perform the analysis as per the standard operational guidelines/ manuals of the instrument. Following analysis perform the washing step of the equipments. The surface of the autoanalyzer must be disinfected with 70% ethyl alcohol. Disinfect and clean the working area before leaving with 0.5% Hypochlorite or 70% Ethyl alcohol.^26–27^

## WASTE DISPOSAL

After the completion of the analysis, the sample should be discarded with open cap in a plastic bucket containing 1% hypochlorite for one hour.^5^ The pretreated vacutainer should be discarded in a double layered plastic bag with tip up. The bag should be disinfected with 1% hypochlorite and safely discarded in the infectious waste container. The PPE should be doffed and disposed in the plastic bag as an infectious waste. The final waste should be disposed by incineration.

## BEFORE LEAVING THE LABORATORY

The laboratory area concerned with sample sorting, labelling, separation must be properly disinfected by 0.1% Hypochlorite, a minimum of 62%-71% ethanol, 0.5% hydrogen peroxide ammonium or phenolic compounds. Centrifuge must be cleaned with 70% alcohol or alcohol-based disinfectant Hand hygiene must be rigorously practiced.^21,26^

## STANDARD PRECAUTIONS

In general, standard precautions are the minimum infection prevention practices that apply to all patient care, regardless of suspected or confirmed infection status of the patient, in any setting where health care is delivered. These practices are designed to protect laboratory personnel and prevent the laboratory personnel from spreading infections among patients.^27^ Standard Precautions in common scenario includes: hand hygiene, use of personal protective equipment (e.g., gloves, masks, eyewear etc.), respiratory hygiene / cough etiquette, sharps safety (engineering and work practice controls), safe injection practices (i.e., aseptic technique for parenteral medications), sterile instruments and devices, clean and disinfected environmental surfaces.^27^ For LP, handling specimens of confirmed cases of COVID-19, the two major standard precautions to be strictly abided by are: hand hygiene and use of personal protective equipment (e.g., gloves, masks, eyewear etc.).

## WAYS FORWARD

This biosafety guidelines is intended to provide the basic safety guidelines to the laboratory professionals working in the field of clinical biochemistry and handling the blood and other body fluid samples of COVID-19 patients. It provides an insight for the correct and scientific measures in handling infectious sample of COVID-19 patients, thereby preventing the health hazard to the medical laboratory professionals as much as possible.
